# AFM Imaging of Lipid Domains in Model Membranes

**DOI:** 10.1100/tsw.2003.12

**Published:** 2003-03-17

**Authors:** Pierre Emmanuel Milhiet, Marie-Cecile Giocondi, Christian Le Grimellec

**Affiliations:** Centre de Biochimie Structurale, CNRS UMR 5048-Univ. Montpellier I, INSERM U554, 29 rue de Navacelles, 34090 Montpellier Cedex, France

**Keywords:** membrane lipids, lateral heterogeneity, monolayer, bilayer, rafts, DRMs

## Abstract

Characterization of the two-dimensional organization of biological membranes is one of the most important issues that remains to be achieved in order to understand their structure-function relationships. According to the current view, biological membranes would be organized in in-plane functional microdomains. At least for one category of them, called rafts, the lateral segregation would be driven by lipid-lipid interactions. Basic questions like the size, the kinetics of formation, or the transbilayer organization of lipid microdomains are still a matter of debate, even in model membranes. Because of its capacity to image structures with a resolution that extends from the molecular to the microscopic level, atomic force microscopy (AFM) is a useful tool for probing the mesoscopic lateral organization of lipid mixtures. This paper reviews AFM studies on lateral lipid domains induced by lipid-lipid interactions in model membranes.

## INTRODUCTION

Whereas the asymmetrical distribution of phospholipids and glycolipids between the exoplasmic and cytoplasmic leaflets of plasma membranes was clearly established in the 1970s[1], the in-plane organization of membrane constituents remains poorly understood. For the last 25 years, the existence of lipid microdomains, i.e., areas that differ in lipid composition from other areas in the membrane in the absence of structural diffusion barriers, was the object of intensive research and animated debates among membrane biophysicists, biochemists, and biologists[2,3,4,5,6].

Evidence for the presence of a category of microdomains enriched in sphingolipids (SL) and cholesterol (Chl), designed as lipid rafts, has been accumulating over the past several years[7,8,9]. The wide interest for rafts came from their potential role in various cell functions including the establishment and maintenance of cellular polarity, signal transduction processes, infection by pathogens like HIV, internalization of toxins. Some evidence also suggests that rafts may be involved in prion and Alzheimer's diseases[10,11,12]. Glycosylphosphatidyl-inositol-anchored (GPI) proteins, doubly acylated proteins such as Src-family kinases or the $\alpha$-subunits of heterotrimeric G proteins as well as transmembrane proteins, particularly palmitoylated ones, show affinity for rafts which can selectively incorporate or exclude proteins to variable extents[10,11,12].

*In situ* detection of membrane microdomains is essentially achieved by a combination of microscopy based techniques like fluorescence recovery after photobleaching (FRAP), fluorescence resonance energy transfer (FRET), single molecule fluorescence microscopy (SMFM), single particle tracking (SPT), laser optical trap (LOT), photonic force microscopy (PFM), and of methods modifying either the lipid composition of membranes, using for example the Chl sequestering agent methyl-$\beta$-cyclodextrin, or the aggregation state of some membrane proteins, often via antibody crosslinking[12,13]. Using these different tools, the size of membrane microdomains varies between a few tens of nanometers to micrometers[13]. Information on the biochemical composition and the physical properties of rafts essentially comes from the analysis of membrane domains resistant to nonionic detergent extraction in the cold (DRMs). This assumes that DRMs correspond to aggregated rafts and, more generally, that there is a close relation between rafts and DRMs[10,11,12]. Determination of the lipid composition of the first DRMs, extracted from MDCK cells by Triton X-100, led to a SL/Chl/glycerophospholipids (GPL) molar ratio close to 1/1/1[14]. With this Chl concentration, DRMs membrane lipids are likely to be in a liquid ordered phase ($l_o$) or a state with similar properties (Fig. 1). The $l_o$ phase is formed by the interaction of phospholipids with Chl[15,16,17,18]. It is characterized by a high degree of acyl chains order associated with lateral diffusion properties close to those determined for lipids in the liquid-crystalline or fluid phase ($L_\alpha$ or $l_d$ for lipid-disordered) where the acyl chains are kinked and loosely packed. For lipids in gel phase, acyl chains are even more ordered than in the $l_o$ phase but the lateral diffusion is two orders of magnitude slower. According to the current view, the formation of rafts is driven by a $l_o$-$l_d$ phase separation process in which $l_o$ SL/Chl enriched lipids domains are surrounded by a $l_d$ matrix enriched in more unsaturated GPL species. Natural sphingomyelin (SM), the most abundant SL in membranes, offers the peculiarity to undergo very broad gel to liquid crystal phase transitions that can extend from below 20°C up to 55°C and thus include the physiological temperature[19]. In biological membranes, they are essentially localized on the exoplasmic leaflet, which strongly suggests that this membrane leaflet plays a crucial role in the existence of rafts.

As pointed out in several recent reviews, many questions like the size(s), the kinetics of formation and lifetime, the organization of the cytoplasmic leaflet facing raft and its coupling with the exoplasmic leaflet remain open[11,12,13,20]. This is not surprising, considering that many of these questions are still unanswered in artificial membranes made of lipid mixtures. As mentioned above, the probable size range of rafts, from a few tens of nanometers up to micrometer, is a major obstacle to their topographical observation with usual optical microscopy techniques.

Atomic force microscopy (AFM), also known as scanning force microscopy (SFM), can image the surface of nonconducting samples in vacuum, air, or in aqueous solutions[21]. This is achieved by using a sharp tip which raster scans the surface of samples adsorbed on a solid support, keeping minimal the force applied to the sample by the tip. Topographic details of biological samples in physiological solutions can be acquired with a lateral resolution better than

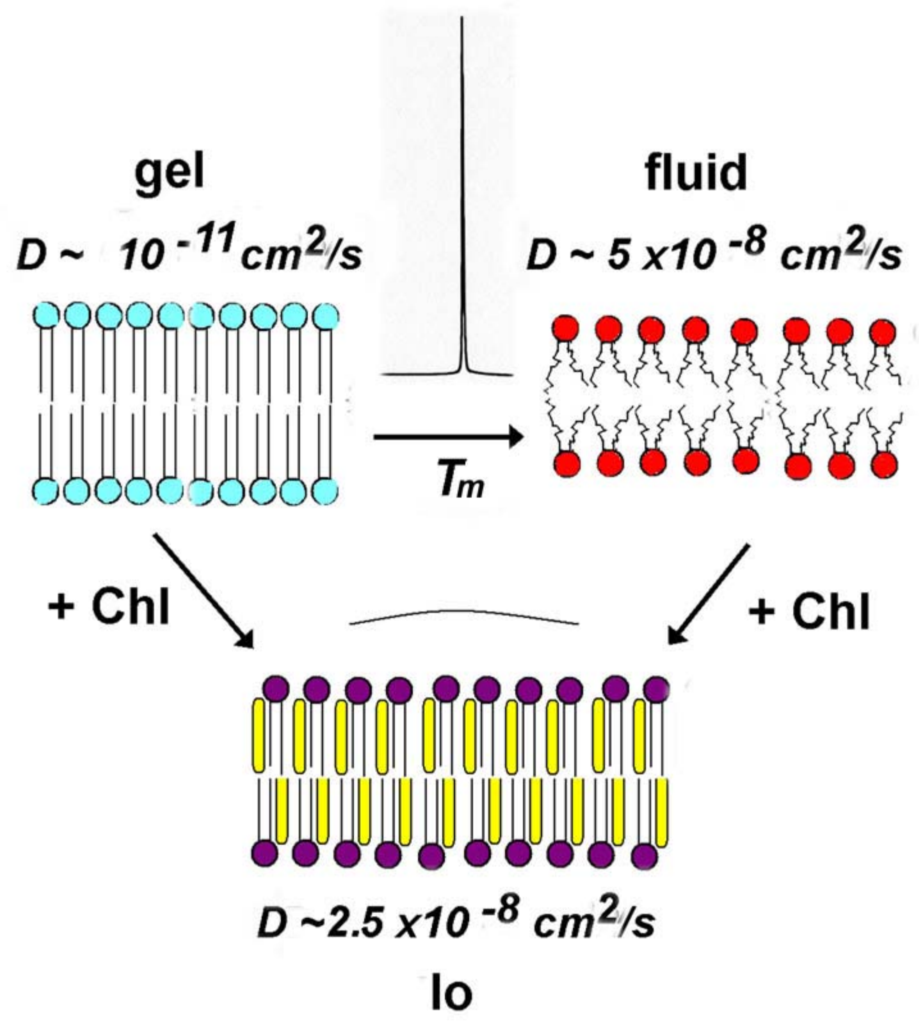

**FIGURE 1**. Phase behavior of phospholipids. In the presence of aqueous buffer, a majority of membrane phospholipids form spontaneously solvated lipid bilayers that can exist in two distinct physical states, gel and fluid, according to the temperature. In the gel phase, molecules are tightly packed in a quasi-hexagonal array with their extended and ordered fatty acid chains lying parallel to each other. Intra- and intermolecular motions are slow, with lateral diffusion coefficient $D < 10^{-11} cm^2/s$. In the fluid phase, acyl chains are highly mobile and the molecules undergo fast rotational and lateral (D) diffusion. This is accompanied by a thinning of the bilayer. For pure phospholipid species, the fluid to gel transition is characterized by a melting temperature, Tm, recorded as a sharp peak by differential scanning calorimetry. Upon addition of cholesterol (20–25 mol% for mono- and disaturated phosphatidylcholines), the sharp peak is abolished and the lipid bilayer is in a liquid ordered ($l_o$) phase. In the $l_o$ phase, the acyl chains are ordered and relatively extended but the molecules have a high rotational and lateral mobility.

0.6 nm and a vertical resolution of about 0.1 nm[22,23,24]. Because of these performances, AFM was applied earlier to the structural characterization of self-assembled or Langmuir-Blodgett (LB) films[25,26]. As recently reviewed, AFM applied to lipid mixtures of biological interest[27,28,29] also provided new information, inaccessible to other techniques, concerning the topographic organization in artificial membranes. In this paper, we will focus on the results obtained on lipid model membranes under ordered-disordered phase separation, an important approach to get more insights in the formation of domains in biological membranes.

## AFM Imaging of Lipid Domains in Monolayers

LB films have been extensively used to model biological membranes and have provided invaluable information on lipid-lipid and lipid-protein interactions in these membranes[30,31,32]. They allow us to define the properties of each membrane leaflet independently of the possible influence of the other leaflet. This is an important point because little is known about the eventual coupling between membrane leaflets. To form films with phospholipids or lipid mixtures that can include also peptides or proteins, lipids are dissolved in a volatile solvent that is spread at the air-water interface (Fig. 2A). After solvent evaporation, the monolayer is compressed, giving surface pressure vs. surface area isotherms characteristic of the monolayer physical state. In monolayer studies, the liquid expanded (LE) state corresponds to lipids that remain in a state equivalent to the $L_\alpha$ in bilayer all along the compression, whereas the liquid condensed (LC) state would correspond to lipids in the gel phases.

Fluorescence microscopy is commonly used to study the morphology and phase behavior of large domains in monolayer (Langmuir) films, but the lateral resolution does not give access to the mesoscopic scale[33,34]. Much higher resolution imaging of monolayers is now possible with AFM. However, AFM imaging requires that the film be transferred to a solid substrate (LB film), at a chosen surface pressure, a procedure that might affect the monolayer organization. Comparison of phospholipid films under phase separation labeled with fluorescent probes indicated that, at microscopic scale, the monolayer topology is retained when the transfer is accomplished at pressures greater than 10 mN/m[35]. When modeling biological membranes, transfers for AFM experiments are generally performed between 30 and 40 mN/m, a surface pressure range considered as a good approximation of the situation found in biological membranes[36,37]. Both the reorganization at low surface pressure and the maintenance of the film structure at high pressure were recently confirmed[38,39]. The main difference between Langmuir films at the water-air interface and LB films examined in air is that, once transferred onto the support, the LB film phospholipids do not diffuse laterally. AFM studies on LB films of biological interest have generally been performed using mica that, once freshly cleaved, gives a very flat hydrophilic surface as a support. When transferring the monolayer to the mica surface, the lipid polar head groups face the mica surface leaving the hydrophobic tails exposed to air (Fig. 2B). On such samples, AFM is therefore performed in air and probes the topography of the hydrophobic part of the monolayer. Data on lipid monolayers fall in two categories: those examining the properties of single species or binary mixtures of common phospholipids and those more specifically related to rafts constituents.

### *Studies of Single Species or Binary Mixtures of Phospholipids*

The AFM images of films made of a single phospholipid species, in a single phase either gel or $L_\alpha$, reveal a homogeneous surface of low roughness (Fig. 2D). In contrast, when the film is under LE-LC phase-separation conditions, the domains formed by all *trans* fully extended acyl chains of the LC phase protrude from the LE fluid matrix, which allows their detection by AFM[40]. This is the case, for example, of dipalmitoylphosphatidylcholine (DPPC) films transferred at 9 mN/m[41] or of natural SMs transferred at 30 mN/m[42] (Fig. 3A). In the case of DPPC, besides large (>10 μm in diameter) LC domains, AFM reveals small mesoscopic domains nondetected by far field fluorescence microscopy images of the same preparation. The lateral distance between the LC domains of SM is also below the limit of fluorescence detection, which implies that this

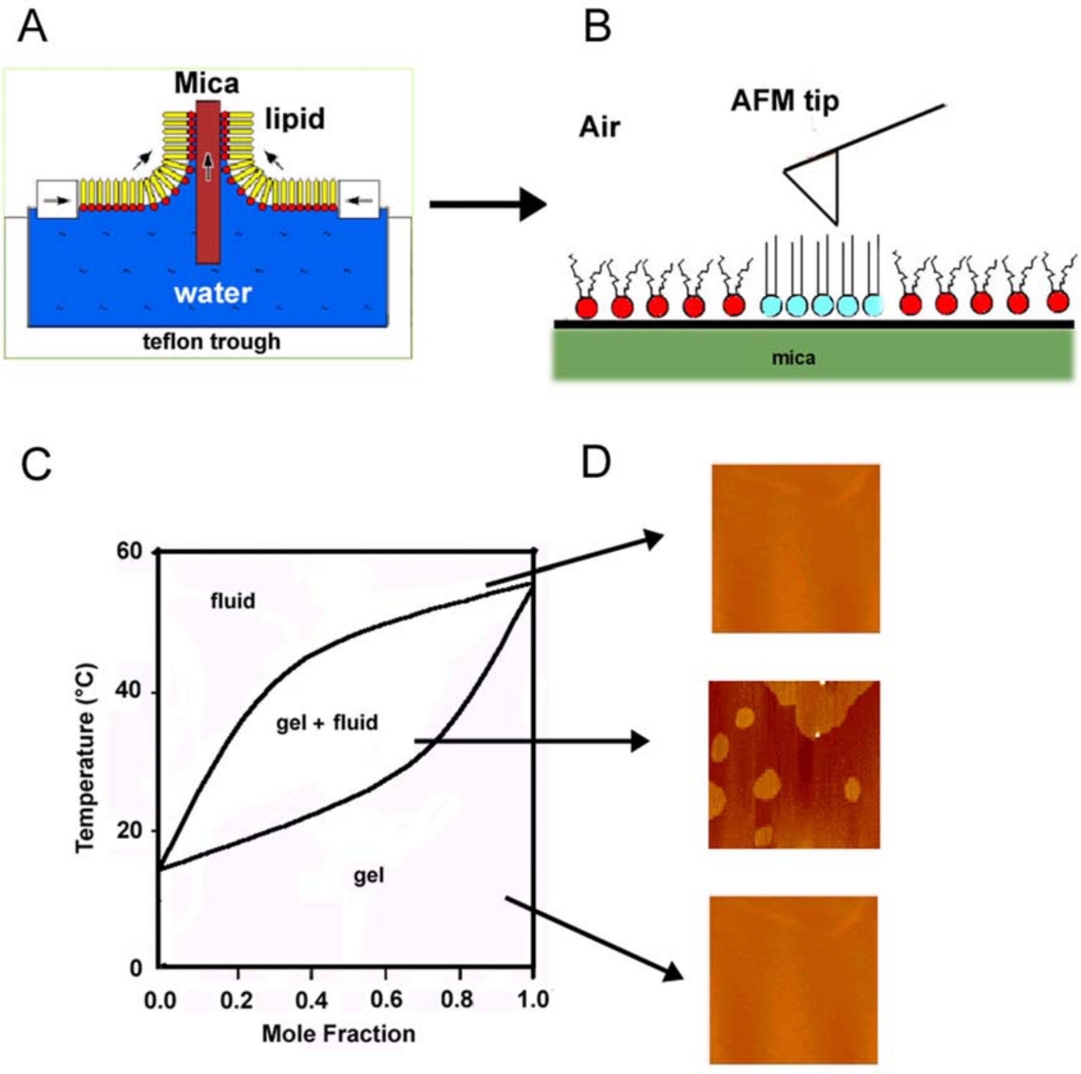

**FIGURE 2**. Running AFM experiments on lipid film. (A) After spreading of lipids at the air/water interface of a Langmuir trough, the film is compressed up to the chosen surface pressure by two Teflon barriers and transferred onto a freshly cleaved mica piece; (B) the sample is placed under the AFM tip and examined in air, the methyl end of lipids in the ordered phase (LC, blue) protrudes from the fluid (red, LE) matrix; (C) hypothetical phase diagram showing the presence of an ordered/fluid coexistence region; (D) AFM representative images of one-phase films (top and bottom) and ordered-fluid phase separation region (middle). The protruding ordered phase domains appear as lighter zones surrounded by a darker fluid matrix.

phase-separated sample would appear homogeneous by optical microscopy. In the DPPC experiments, LC domains protrude by ~5–8 Å from the surrounding LE regions. Such a value is slightly higher than expected from the thickness difference between the gel and fluid state of DPPC bilayers recorded by neutron diffraction[43]. This is likely due to a contribution, in the topography images, of the respective mechanical properties of the two phases[44]. When imaging in air under ambient conditions, due to the presence of a thin water film, strong adhesion forces between the tip and the films impose the use of scanning forces significantly higher than those required for imaging under liquid. For SM samples, the LC domains protruded from the LE matrix by ~1 nm (Fig. 3B), an observation which can be explained by the fact that the shortest or unsaturated acyl chains are melting first, thus enhancing the difference in height between the LE

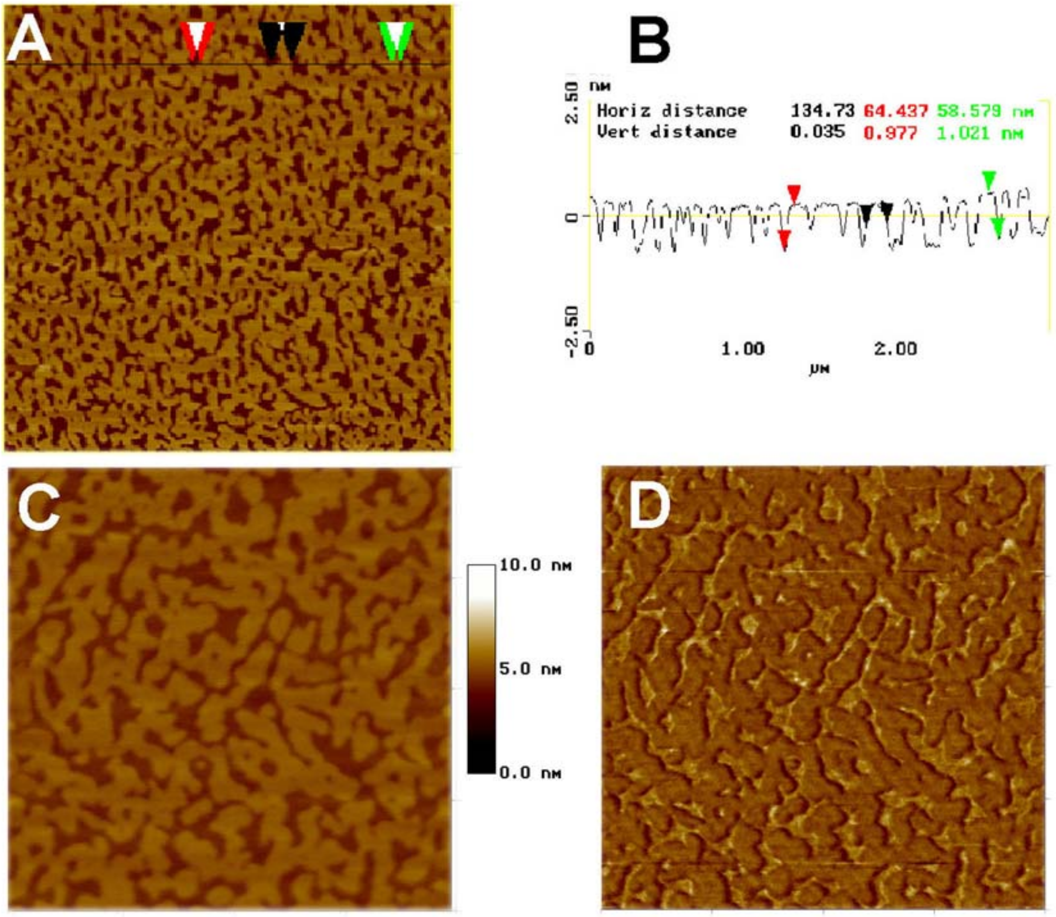

**FIGURE 3**. AFM imaging of bovine brain SM transferred at 30 mN/m. (A) Height image of SM, scan size 3 × 3 µm; (B) virtual sectioning corresponding to the horizontal black line drawn in A. Ordered (lighter) domains protrude by ~1 nm (red and green arrows) from the fluid matrix. Width of the ordered domain marked by dark arrows is ~135 nm; (C) height image at a higher magnification (scan size 1 × 1 µm). The color scale on the right gives the relative height of structures; (D) friction image obtained simultaneously.

and LC phases. As illustrated by these two examples, most studies on the characterization of lipid domains by AFM rely on the difference in apparent thickness between domains under different phases. Frictions forces between the tip and the sample (Fig. 3D), as well as tip energy dissipation which reports on viscoelastic properties of samples when the AFM is running under an oscillating mode, can also be used to reveal the existence of phase separations[44,45]. This has been applied to LB films made of various phospholipid binary mixtures in the region of coexisting phases, including dioleoylphosphatidylethanolamine (DOPE)/dipalmitoylphosphatidylethanolamine (DPPE)[45], DOPE/distearoylphosphatidylethanolamine (DSPE)[44], and dioleoylphosphatidylcholine (DOPC)/dipalmitoylphosphatidylcholine (DPPC)[46] (Fig. 4A) as well as of binary and ternary mixtures that mimic the phase behavior of stratum corneum lipids[47]. These studies have established the usefulness of AFM for the detection of membrane domains ranging from the nanometer to the micrometer scale in supported monolayers made of lipid mixtures.

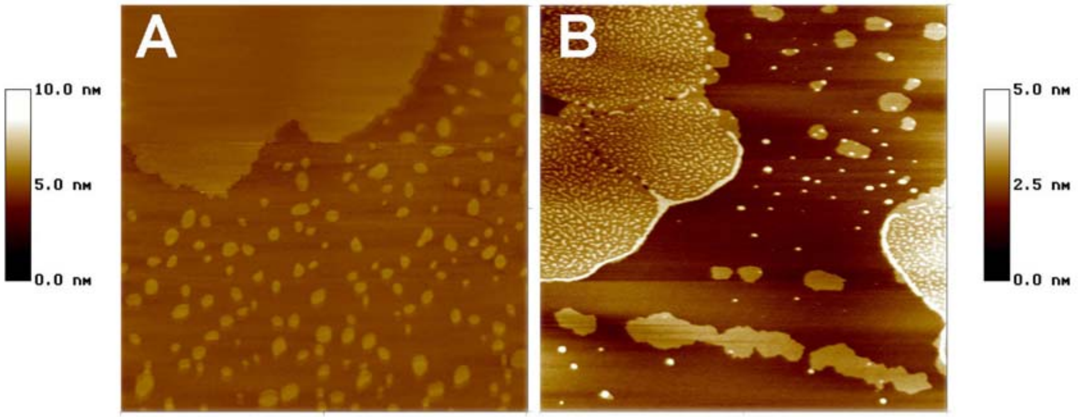

**FIGURE 4**. $GM_1$ microdomains formation in phase-separated DOPC/DPPC films. (A) Both large and small ordered domains are observed in 1:1 DOPC/DPPC films transferred at 30 mN/m (scan size 4 μm); (B) addition of 4 mol% GM1 induces the formation in the ordered phase of small microdomains which can fuse to form fencelike structures at the large domains/matrix boundary (scan size 2 × 2 μm).

## *Studies of Lipid Mixtures Related to Rafts Constituents*

Because the presence of both SL and Chl in the exoplasmic membrane leaflet appears to be determinant for the existence of rafts, it is crucial to define how lipids interact with each other, and with intercalated proteins, in order to understand the principles behind the dynamics of the cell membranes. Among SL, glycosphingolipids (GSL) are constituents of rafts which act as receptors for numerous biologically active agents. Determination, at the mesoscopic scale, of their distribution in phase-separated lipid mixture by indirect methods has led to conflicting results[48,49,50]. This was the case for one of the most studied representatives of mammalian ceramide–based GSL, the $G_{M1}$ ganglioside, which is the natural receptor for cholera toxin. Direct AFM examination of monolayers doped by low concentrations of $G_{M1}$ (<10%) have shown that this ganglioside is immiscible with either LE DOPC or ordered LC DPPC[46]. Thus, upon addition of $G_{M1}$ to DPPC, patches 15–30 nm in diameter and filamentous structures protruding by ~1 nm above the DPPC acyl chains methyl end are observed, whereas $G_{M1}$ forms large (LC) domains that emerge by ~2 nm from the DOPC surface. When added to the phase separated mixture of DOPC/DPPC, $G_{M1}$ also formed round shaped and filamentous microdomains, preferentially localized in the LC phase DPPC domains, which eventually fused to form fence-like structures at the LE/LC interface (Fig. 4B). The presence of $G_{M1}$-enriched microdomains in large DPPC domains indicated the existence of a LC/LC immiscibility, most likely driven by hydrophobic mismatching. A similar behavior of $G_{M1}$, with the formation of both rounded and filamentous microdomains, was also observed in 2:1 DPPC/Chl monolayers for which a physical state equivalent to the $l_o$ phase is expected[51]. This suggests that small glycosphingolipids-enriched microdomains can exist within larger ordered domains.

The behavior of SM/Chl-enriched domains as a function of their Chl content has been studied[52] in a model of the exoplasmic leaflet of renal brush border membranes (BBM). The phospholipid composition of renal BBM exoplasmic leaflet is ~75% SM and 25% zwitterionic phospholipids, essentially PC[53]. In contrast to intestinal BBM, the renal BBM from the proximal tubule show a very low content in glycosphingolipids[54]. Monolayers made of SM and palmitoyloleoylphosphatidylcholine (POPC), at molar ratios varying from 2:1 to 4:1, were phase-separated into LC SM-enriched phase and LE POPC-enriched phase (Fig. 5B). At a 2:1 molar

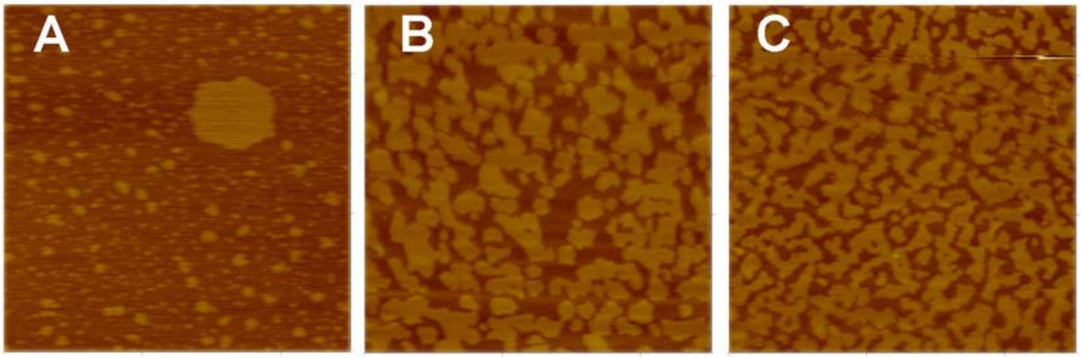

**FIGURE 5.** Microdomains in SM/POPC monolayers (30 mN/m). SM enriched ordered microdomains in 1:3 (A) and 3:1 (B) SM/POPC monolayers. (C) 3 :1 SM/POPC + 20 mol% cholesterol. Scan size 2.5 × 2.5 µm.

ratio the size of LC domains varied between 50 and 200 nm, with an average area of ~1 × $10^4$ $nm^2$, whereas for a 4:1 molar ratio the mean size of domains was increased three times. These data strongly suggest that, even in the absence of cholesterol, lipid microdomains may exist in the exoplasmic leaflet of renal BBM and, by extrapolation, in the exoplasmic leaflet of sphingolipids rich cells. In fact, Chl promoted the connection between SM domains at 20 mol% (Fig. 5C), then progressively reduced the size of domains and the height differences between the phases up to 33 mol%. For this Chl concentration, the monolayer was still heterogeneous and consisted of a «network» composed of small (20–70 nm), likely $l_o$ branched domains emerging from the matrix by ~0.4 nm. Both for the distribution of $G_{M1}$ between LE and LC phases and for the model of BBM exoplasmic leaflet, lipid domains would have escaped optical microscopy detection due to their small size. For most cell types the SM content of the plasma membrane is significantly lower than that determined in renal BBM. Bilayers made from 1:3 SM/POPC mixtures, which would correspond to a total SM content of ~10–15 mol% in the plasma membrane, i.e., a value commonly found in many cell types, also show the presence of small LC microdomains dispersed in the LE matrix (Fig. 5A). This observation supports the hypothesis that LC/LE lipid phase separation may occur in the plasma membrane exoplasmic leaflet in the absence of Chl. In a recent paper, Dietrich et al. have examined by fluorescence microscopy the organization of monolayers made either of POPC/SM/Chl (2:1:1), DOPC/SM/Chl (1:1:1), or total lipids extracted from renal BBM[55]. They reported the presence of $l_o$ domains much larger, i.e., several micrometers in diameter, than those observed for renal BBM models. This difference in the size of $l_o$ domains between the two series of experiments most likely resulted from the use of monolayers with both different phospholipid molar ratios and different preparation methods. Thus, in the fluorescence microscopy experiments, the monolayer was transferred on silanized glass and examined in buffer. In contrast with LB film deposited on mica, lipids diffuse laterally in monolayers deposited on alkylated substrata, and the size and shape of domains was found to vary considerably between experiments[55]. Moreover, for renal BBM, lipids extraction results in the dilution of lipid present in the external leaflet by those of the inner leaflet. Because single membrane leaflet properties might be affected by the presence of the other membrane leaflet, the phase-separation properties of supported bilayers have been also investigated by AFM.

## AFM Imaging of Lipid Domains in Supported Bilayers

AFM imaging of supported lipid bilayers in buffer has been performed using both contact and oscillating modes. Successive transfer of two monolayers and vesicles fusion are the principal methods for preparing supported bilayers. The double transfer uses either the LB or the Langmuir Schaefer (LS) techniques. Following the formation of the first mica-facing LB film as described above (inner leaflet), the second monolayer (outer leaflet) is transferred by either vertical (LB) or horizontal dipping through the monolayer at the air-water (buffer) interface, resulting in the exposure of these second monolayer polar head groups to the aqueous buffer. In the vesicles fusion method, a suspension of small unilamellar vesicles is deposited on freshly cleaved mica. After interaction with the support, vesicles spontaneously form a bilayer, a process that can be followed by AFM in real time[56]. The advantage of the double transfer methods is that asymmetrical bilayers can be formed. However, thinning of the water layer between the mica and the inner leaflet, during the lag-time before the second monolayer transfer, often results in change in the diffusion properties of this inner leaflet and eventually "freezes" it[41]. On the other hand, vesicle fusion leads to symmetrical bilayers but a buffer layer ~1–3 nm in thickness separates the bilayer from the substrate, which insures that these supported bilayers do have the essential feature of free-standing lipid bilayers[57,58,59].

### *External Leaflet Topography of Bilayers Made by Transfer*

The behavior of phase-separated lipid mixtures in asymmetrical bilayers has generally been investigated with an inner leaflet made of a gel phase PC (DPPC) or PE, either DPPE or distearoyl-PE (DSPE), which enhances the bilayer stability[28,60,61,62,63], although egg-PC has been occasionally used[55]. The outer leaflet was made of binary mixtures of DPPC/POPC[64], DOPE/DSPE[44], or DSPE/monogalactosylethanolamine (MGDG)[65]. In all cases, large (~1.5–10 µm) gel phase domains of various shapes were observed at the surface of the outer leaflet. Direct comparison between DOPE/DSPE monolayer and DSPE supported DOPE/DSPE bilayer showed that gel DSPE-enriched domains, albeit of similar size, have a more complex elongated shape in the bilayer[65]. This change in shape may originate from repulsive interactions between individual electric dipoles[44]. Calcium-induced phase separation, still leading to domains larger than 500 nm, was reported for POPC/palmitoleoylphosphatidylglycerol (POPG)[23] and for DPPC/dipalmitoylphosphatidylserine (DPPS)[66] outer leaflets. Although the apparent thickness differences between gel and $l_d$ phases can be function of the scanning force[23,44,65], in such asymmetrical bilayers the gel phase protruded from the $l_d$ phase by a single value. This contrasted with bilayers for which the deposited inner monolayer was under LE/LC phase-separation: under this condition, the AFM image of the bilayer exhibited discrete height changes in the film topography that occured in multiples of ~0.8 nm, approximately equal to the LE/LC height difference determined in the monolayer. Three quantized height levels were observed, consistent with stacking of LE on LE, LE on LC (or LC on LE) and LC on LC domains[41]. Simultaneous observation of the same samples by scanning near field optical microscopy (NSOM) using the fluorescent probe $diIC_{18}$ which preferentially labels the LE phase, strongly suggested that in such bilayers the inner and outer leaflets behaved independently, i.e., were uncoupled. In accordance with data obtained on LB films, $G_{M1}$ promoted the formation of domains 30–200 nm in diameter in bilayers made of a pure DPPE inner leaflet and of DPPC or 2:1 DPPC/Chl outer leaflet[63]. To summarize, AFM images in buffer of the hydrophilic interface of phase-separated supported bilayers made by the successive transfers of two monolayers are in general agreement with those obtained in air on LB films. In these samples, gel-phase domains are large and the two membrane leaflets uncoupled.

## Bilayers Made by Vesicle Fusion

The presence of a significant buffer layer between the support and the bilayer has allowed us to investigate both the basic properties of the phase separation process in phospholipid binary mixtures and the behavior of lipid mixtures related to rafts constituents.

The kinetics of microdomains growth, their size, and the coupling between membrane leaflets were studied in DOPC/DPPC (1:1 and 3:1) supported bilayers[67]. In accordance with theoretical prediction[68], it was demonstrated that the complexion of the phase separation process can take hours following a temperature quench from the one-phase fluid region to the gel-fluid phase region. A similar conclusion was recently reached applying fluorescence techniques to large unilamellar vesicles[69]. These experiments also showed that the gel phase separation proceeded by a slow growth of individual gel-phase domains, instead of by an increase in their number as proposed for LigGalCer/DPPC mixtures[70]. The growth process was characterized by an algebraic growth law and the evolution of individual gel domains suggested that, in the transient regime, the growth was governed by a ripening mechanism. The exponent value (0.66) was however about twice that predicted by computer-simulation techniques[68]. In these experiments, only two quantized height levels were observed in topographic images. This observation strongly suggested that, in contrast to bilayers made by transfer of monolayers, the inner and outer membrane leaflets were coupled with gel domains exactly superimposed upon each other in the two monolayers. Furthermore, time-dependent examination of the growth of single gel microdomains indicated that they were growing simultaneously at the same sites in each leaflet of the bilayer as they remain superimposed on each other throughout the phase separation process[67]. Coupling between leaflets was observed in AFM topographic images of various lipid mixtures, like DPPC/POPC (Fig. 6A)[64], DMPC/DSPC[71], POPC/SM[72], DOPC/SM (Fig. 5B)[73,74, however see 75], DLPC/DMPS[76], and DPPC/DOPS[77] under gel-fluid phase-separation. Phase coupling between the two leaflets was previously observed using FRAP on DMPC/DSPC multibilayers[70] and, more recently, in giant liposomes using fluorescence techniques[55,78]. AFM temperature dependent experiments also brought direct evidence for the transition from the disconnected fluid to the disconnected gel region of the phase diagram for DMPC/DSPC bilayers[71]. It is worth noting that, according to the experiment, the temperature at which the gel to fluid phase transition occurred was found to correspond to[71] or to be shifted 3–4°C upward[79] as compared to that determined on multilamellar vesicles by differential scanning calorimetry. This is most likely due to variations in the thickness of the buffer layer between the mica and the bilayer, a parameter that is poorly controlled when preparing supported bilayers.

Except for one report[80], the average size of ordered microdomains in phospholipid binary mixtures examined so far by AFM is much larger than that predicted from indirect ESR and fluorescence experiments or from Monte Carlo simulations, where only about 500–1000 molecules per gel domain were found using similar lipid mixtures[70,81,82,83]. Thus, domains larger than 1 μm, i.e., containing a number of molecules in the $10^6$ range, were observed in DPPC/POPC[28,64], DPPC/DOPS[77], DMPC/DSPC[71], DPPC/DOPC[67], SM/POPC[72], SM/DOPC[74] binary mixtures. Earliest studies based on the use of freeze-fracture electron microscopy and electron diffraction also described domains in the μm range for liposomes made of various phase-separated binary mixtures of phospholipids[84,85,86,87]. Three additional pieces of information were collected from these AFM studies. First, the size range of the individual gel domains found in a sample could vary by more than an order of magnitude. For example, in 3:1 DOPC/DPPC sample examined at room temperature 2 h after the temperature quench from the miscible state, the size of gel phase domains varied between ~80 nm and 2 μm[67]. Second, as illustrated by Fig. 7, for a temperature-induced phase–separation, the size of the largest gel domains in a given mixture was a function of the thermal history of the sample, an observation already reported in the 1970s[86]. Starting from the miscible state, using a slow

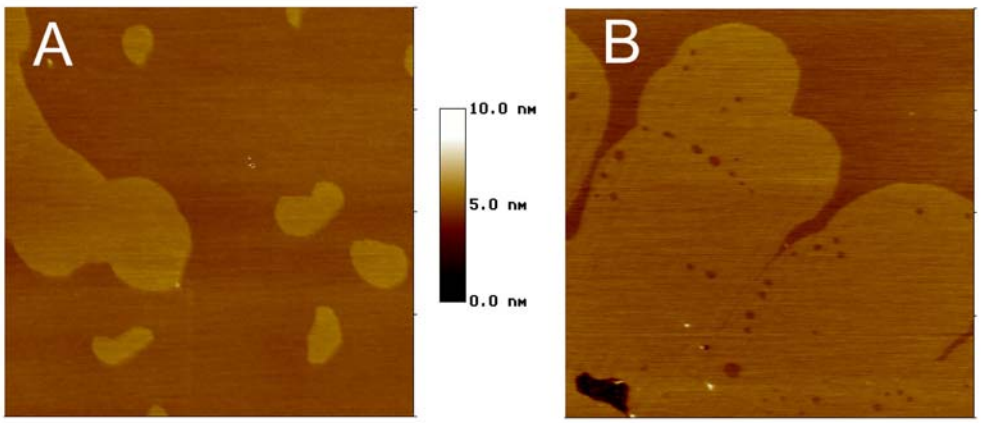

**FIGURE 6**. Imaging of supported bilayers made by vesicles fusion. Phase separation in 1:1 DOPC/DPPC (A) and 1:1 DOPC/SM (B) bilayers examined in phosphate buffer (scan size 4 × 4 µm).

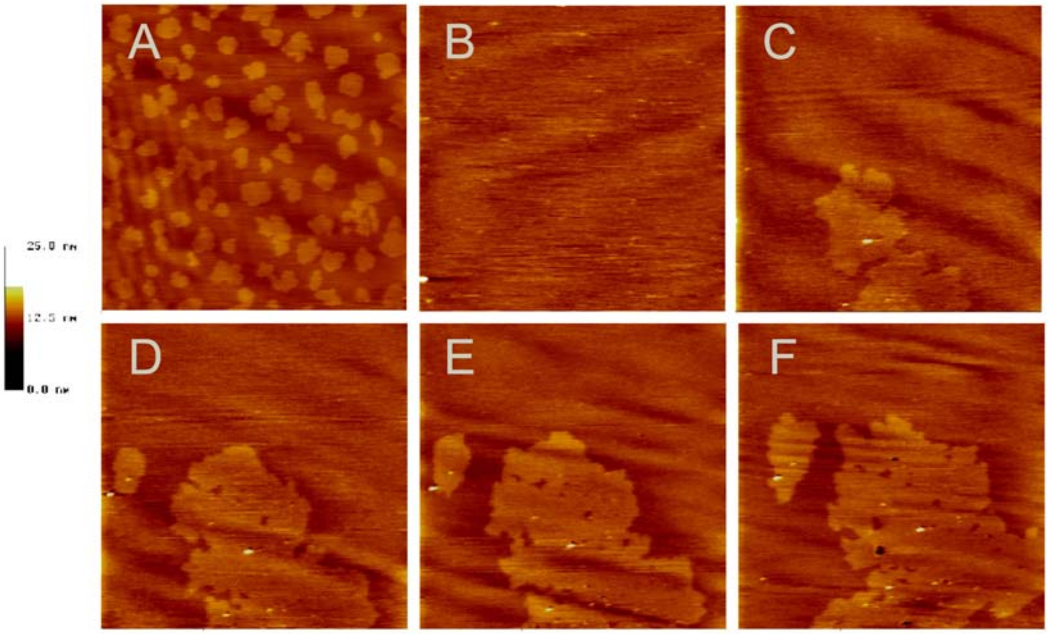

**FIGURE 7**. The size of ordered domains depends on experimental conditions. (A) 1:1 DOPC/DPPC bilayers examined in buffer 120 min after a rapid temperature quench from 60 to 23°C; (B) upon heating of the bilayer to 45°C under the microscope, gel phase microdomains disappear; (C to F) sample temperature was linearly decreased from 45 to 25°C in 2 h, promoting the formation of large domains; (C, D, E, and F) 40, 80, 100, and 120 min (scan size 15 × 15 µm).

decrease rather than a rapid quench in temperature to bring the system in the gel-liquid crystal coexistence region favored the formation of two order of magnitude larger domains, keeping constant the total area of the ordered domains. Finally, addition of calcium to a binary mixture containing a negatively charged phospholipid markedly reduced the size of gel domains[77]. These observations indicate that in a simple phase-separated phospholipid mixture, the size of gel domains can markedly vary according to the experimental conditions. This variability has to be kept in mind when analyzing the actual controversy concerning the size of ordered domains in biological membranes.

In biological membranes, microdomains formation most likely involves a $l_o$- $l_d$ rather than a gel-$l_d$ lipid phase-separation. Addition of Chl to either SM/DOPC or SM/POPC phase-separated mixtures allowed to compare the topological properties of gel-$l_d$ and $l_o$-$l_d$ bilayers[72,75]. In both models, upon Chl addition, a progressive reduction in the height difference between the phases was observed and the two leaflets of Chl containing bilayers were coupled. As observed in monolayers, the presence of 20 mol% Chl in SM/POPC bilayers promoted the connection of protruding domains, likely in the $l_o$ phase. Further increase in Chl level first made the $l_o$ domain to disconnect (25 mol%) and then to become undetectable (33 mol%), suggesting that at this Chl concentration all the bilayer was in the $l_o$ phase[72]. For SM/DOPC bilayers, the connection between $l_o$ domains was reported to occur at a higher Chl concentration (30 mol%) and the $l_o$-$l_d$ phase-separation was still present at 50 mol% Chl[75]. In accordance with the known physical properties of the $l_o$ phase, successive scans of the same zone revealed modifications in the topology of the bilayer[74]. Furthermore, like in biological membranes, treatment with Triton X-100 in the cold preserved the $l_o$ phase but dissolved the fluid phase[75]. Again, in these studies, the shape and size of ordered domains varied from small (150 nm) disc shaped to large (3 µm) elongated structures.

## CONCLUSION

By giving access to the topology of lipid mono- and bilayers at a mesoscopic scale, AFM allows us to get direct information on the shape, the size, and the kinetics of formation of single domains in gel- and fluid-fluid phase separated mixtures. Most studies were performed on two-component, gel-fluid phases lipid bilayers, the model privileged by biophysicists interested in characterizing membrane lateral heterogeneity. In such systems, the size of ordered domains was generally much larger than that estimated from indirect methods. This was also true for the recently examined ternary mixtures under liquid ordered-fluid phase separation containing Chl and sphingolipids, a situation that better mimics the rafts of biological membranes. So far, only $G_{M1}$ was found to spontaneously form microdomains 30–50 nm in diameter, in the low size range proposed to be that of rafts, in both gel and $l_o$ phases of supported bilayers. However, the data reported in this review clearly indicate that, for a given lipid mixture maintained in the same buffer, whereas the total area occupied at equilibrium by ordered domains is constant, the size of single domains can vary by more than two order of magnitude according to the history of the sample. Besides studies on lipid-lipid interactions, AFM was also recently used to demonstrate the spontaneous insertion of alkaline phosphatase, a glycosylphosphatidylinositol-anchored protein in model rafts[74] and the formation in DOPC/Chl bilayers of cholesterol-rich domains upon addition of NAP-22, a myristoylated protein found in the rat brain DRMs[88]. The objective of all these works on model systems was to better characterize the basic properties of microdomains induced by lipid-lipid immiscibility, the principal mechanism at the origin of rafts in biological membranes. Indeed, this constitutes only a first necessary step in the understanding of rafts structure-function relationships and model systems can be valuable tool for answering many questions like, for example, the organization of lipids and proteins in the rafts inner leaflet. Assays to localize rafts at the surface of living cells using AFM have been unsuccessful up to now **.** The time required for images acquisition and the complexity of the cell topography are two major obstacles against the AFM mesoscopic detection of rafts in living cells, a goal that has been already reached by photonic force microscopy[89]. Attempts to image *in situ* the very fragile

DRMs structures that remain following cell treatment with Triton X-100 and glutaraldehyde fixation have provided encouraging results. Thus, DRMs several micrometers square in size were visualized on CV-1 ghosts. The existence of physical interactions between DRMs and the cytoskeleton was strongly suggested by their respective 3-D organization[90]. Elucidation of the lateral organization of biological membranes remains a major issue in cell biology. The unique character of the topographical data obtained on model systems, at a mesoscopic scale and in physiological conditions, during the last 5 years have established the usefulness of AFM in contributing, in parallel with the other available microscopy techniques, to the understanding of the principles that govern membrane organization. It is likely that, besides topographical information, the opportunity to probe by AFM the local physical properties of membrane via force measurements (nanomechanics, surface forces) will in a near future also significantly improve our knowledge of cell surface microdomains.

## ACKNOWLEDGMENTS

This work was supported by the Ministère de la Recherche, Groupe d'Intérêt Scientifique "Infections à Prions".
